# Bystander Exposure to Ultra-Low-Volume Insecticide Applications Used for Adult Mosquito Management

**DOI:** 10.3390/ijerph8062142

**Published:** 2011-06-14

**Authors:** Collin J. Preftakes, Jerome J. Schleier, Robert K. D. Peterson

**Affiliations:** Department of Land Resources and Environmental Sciences, Montana State University, 334 Leon Johnson Hall, Bozeman, Montana 59717, USA; E-Mails: cjpreftakes@gmail.com (C.J.P.); bpeterson@montana.edu (R.K.D.P.)

**Keywords:** dermal exposure, pyrethroid, risk analysis, exposure assessment, passive dosimetry

## Abstract

A popular and effective management option for adult mosquitoes is the use of insecticides applied by ultra-low-volume (ULV) equipment. However, there is a paucity of data on human dermal exposure to insecticides applied by this method. The objective of the current study was to estimate dermal exposures to the insecticide active ingredient permethrin using water- (Aqua-Reslin^®^) and oil-based (Permanone^®^ 30-30) formulations with passive dosimetry. No significant differences in deposition of permethrin were observed between years, distance from the spray source, front or back of the body, or the placement of the patches on the body. However, exposure to Aqua-Reslin was significantly greater than Permanone 30-30 and average concentrations deposited on the body were 4.2 and 2.1 ng/cm^2^, respectively. The greater deposition of Aqua-Reslin is most likely due to the higher density of the water-based formulation which causes it to settle out faster than the lighter oil-based formulation of Permanone 30-30. The estimated average absorbed dermal exposure for permethrin from Aqua-Reslin and Permanone 30-30 was 0.00009 and 0.00005 mg/kg body weight, respectively. We also found that ground deposition of ULV insecticides can be used as a surrogate for estimating dermal exposure. The estimated exposures support the findings of previous risk assessments that exposure to ULV applications used for mosquito management are below regulatory levels of concern.

## 1. Introduction

A popular management option for adult mosquitoes is the application of ultra-low-volume (ULV) insecticides which have been shown to reduce mosquito density, reduce disease infection rates, and enhance economic benefit by preventing medical and lost work costs [1–7]. Due to rising concerns about global climate change leading to the range expansion of mosquito species that vector human and animal pathogens [8], there has been greater public attention to the human-health and environmental risks associated with ULV insecticide applications [9–11]. Reasonable worst-case risk assessments have been performed in response to concerns about the safety of ULV insecticides. Peterson *et al.* [10] performed a deterministic human-health risk assessment for acute and subchronic exposures to six mosquito insecticide active ingredients, and the synergist piperonyl butoxide (PBO), after ground-based ULV applications. They found that acute and subchronic risks to humans from the insecticides were well below regulatory levels of concern. Schleier III *et al.* [12] performed an acute probabilistic risk assessment of the same insecticides and population groups as Peterson *et al.* [10] further supporting previous findings that the risks to humans from insecticides used for adult mosquito management would most likely not exceed regulatory levels of concern. Both Peterson *et al.* [10] and Schleier III *et al.* [12] used the Industrial Source Complex Short Term version 3 (ISCST3) model to estimate environmental deposition, and therefore also to estimate dermal exposures [13,14]. Schleier III and Peterson [15] demonstrated that ISCST3 overestimated environmental concentrations by as much as 16-fold when compared to actual environmental concentrations. Additionally, Schleier III and Peterson [15] demonstrated that the AGDISP and AgDrift^®^ models were underestimating environmental concentrations, which adds to the uncertainty of past risk assessments [16–18].

Sensitivity analysis, which is the determination of how variation in the output of a model can be attributed to variations in the input assumptions, revealed that the estimated dermal exposure contributed about 41% to the estimated total exposure for adult males and females, youth and children, and about 17% to the total exposure of toddlers and infants [12]. Sensitivity analysis performed by Schleier III *et al.* [12] and Schleier III [19] showed that the estimated inhalation and dermal exposure contributed the most to the model output variance. Schleier III [19] demonstrated that estimated dermal exposure to adult females and males, youth, toddlers, children, and infants using actual environmental concentrations accounted for 85% to 14% of the overall exposure to permethrin after truck-mounted ULV applications.

Currently there has only been one study that examined dermal exposures after ULV applications. Moore *et al.* [20] measured concentrations of malathion using human subjects and found no significant differences between sampling location on the body (torso, arms, legs, and head) at 7.6 and 15.2 m from the spray source [20]. The average concentration of malathion on the chest, arms, legs, and head was 190 ng/cm^2^. Although the majority of deterministic and probabilistic risk assessments using estimated environmental concentrations have not suggested unacceptable exposures, they have relied on models that are designed for industrial plumes and agricultural applications, which greatly differ from ULV applications, to estimate environmental concentrations and thus dermal exposure after truck-mounted ULV applications [15]. Because of the lack of studies examining bystander exposure and the importance of dermal exposure to total exposure, the objective of our study was to estimate dermal exposures after ULV applications of insecticides using passive dosimetry.

## 2. Materials and Methods

Two permethrin formulations (most commonly applied by ULV) were sprayed [21]: an oil-based Permanone^®^ 30-30 (Bayer Environmental Science, Research Triangle Park, NC, USA), and water-based Aqua-Reslin^®^ (Bayer Environmental Science, Research Triangle Park, NC, USA). The water and oil based formulations were chosen for their different densities which can affect movement and deposition [22,23]. Fluorescent tracers were used to quantify the amount of permethrin exposure after ULV applications. Fluorescent tracers have been used to estimate the concentrations of pesticides in spray drift and efficacy studies, and for determining the amount of pesticide that settles onto the target area [24–35]. The oil-soluble tracer Tinopal OB (BASF Corp., Florham Park, NJ, USA) was mixed with Permanone^®^ 30-30, at a rate of 12 g/L and the water-soluble tracer Fluorescein (Aqua Solutions, Deer Park, TX, USA) was mixed with Aqua-Reslin^®^ at a rate of 14 g/L. The addition of fluorescent tracers to pesticide formulations does not alter the density, viscosity, or droplet spectrum of ULV insecticides [22]. Permanone 30-30 was mixed 1:2:1 with Crystal Plus 70T light mineral oil (STE Oil Company, Inc., San Marcos, TX, USA) and American Chemical Society (ACS) grade toluene (99.5% purity, Mallinckrodt Baker, Inc., Phillipsburg, NJ, USA) and applied at a maximum flow rate of 192 mL/min. Aqua-Reslin was mixed 1:1 with deionized (D.I.) H_2_O and applied at a maximum flow rate of 192 mL/min. Both Aqua-Reslin and Permanone 30-30 (20 and 30% permethrin by weight respectively) were applied at the maximum application rate of 7.85 g/ha of permethrin. Between each spray replication the nozzle, pump, and hoses were rinsed with 300 mL of D.I. H_2_O followed by 300 mL of a 1:1 mixture of high pressure liquid chromatography acetone (99.7% purity; EMD Chemicals, Gibbstown, NJ, USA) and ACS grade toluene to reduce cross contamination.

The field site was located in Southwest Montana (45°38′45.76″N, 111°23′45.16″W) and applications occurred between 7 July 2009 to 5 August 2009 and 2 August 2010 to 12 August 2010. No more than three applications were performed for any given formulation per night, and applications began no earlier than 18:00 h Mountain Standard Time. A truck-mounted Guardian 95 ES ultra-low-volume sprayer (ADAPCO, Sanford, FL, USA) cold fogger with a spray pressure of 10 Kpa was used. The sprayer nozzle was oriented at 135˚ with respect to the ground and the truck was driven at 16.1 km/h perpendicularly to the wind direction. Wind direction and speed were recorded by a HOBO^®^ micro weather station (Onset Computer Corporation, Bourne, MA, USA) consisting of a temperature gauge, relative humidity (RH) sensor, and anemometer sensor, and was located upwind of the spray zone. The average wind speed, temperature, and relative humidity for all applications were 213 cm/s, 19 °C, and 48%, respectively. A DC-III portable droplet measurement system (KLD Labs, Inc., Huntington Station, NY, USA) was used to measure the volume median diameter (VMD). The average VMD for Permanone 30-30 and Aqua-Reslin was 21 and 19 μm, respectively.

**Figure 1.**
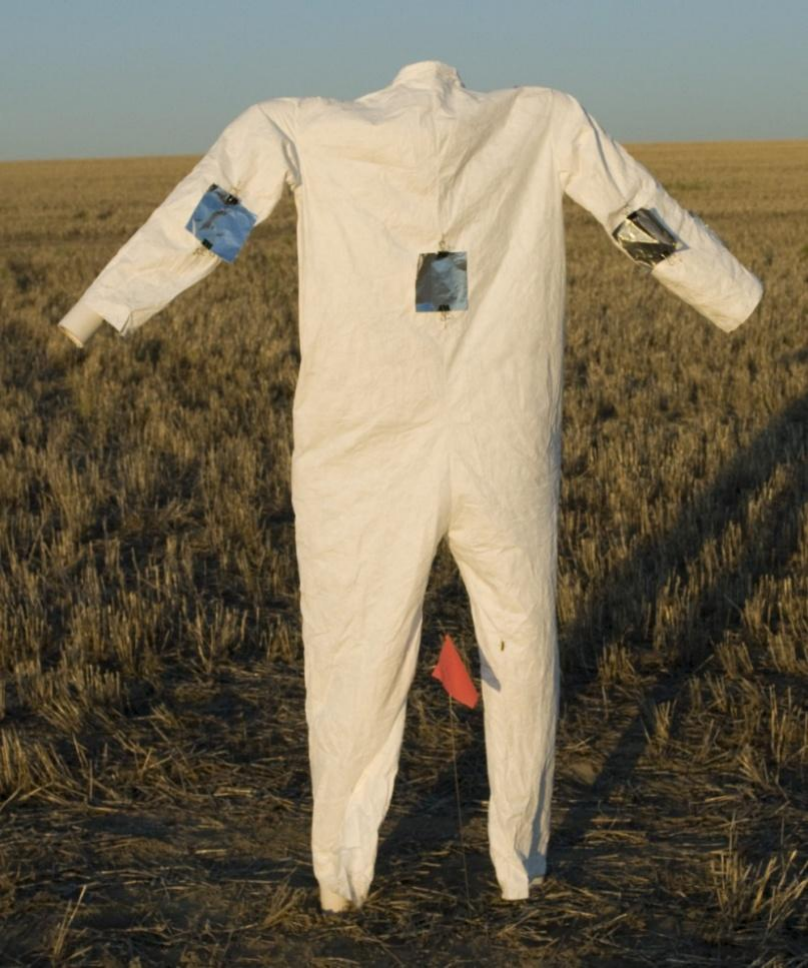
Mannequin bystander dressed Tyvek^®^ disposable coverall suit with one aluminum sampling patch on each arm and leg, upper chest, groin, and center of the back of each mannequin (only shown are the patches on the arms and chest) (photo: © 2009 R.K.D. Peterson).

Two mannequins were used as surrogates for human bystanders to measure deposition at two different distances from the spray source. One mannequin each was placed 25 and 50 m from the spray source at each application site. Mannequins were constructed from 50.8 mm PVC pipe and measured 160-cm tall (no head due to small surface area relative to rest of body [36]) and 45.72 cm from shoulder to shoulder (Figure 1). Tyvek^®^ disposable coverall suits (large size; Figure 1) were used to dress the mannequins and provide a backing for the collection patches. Insecticide deposition was collected on 121 cm^2^ square aluminum foil patches (Figure 1) [37]. Two binder clips were used to attach the aluminum patches to the mannequins. Patches were placed on the outer suit only and located where the greatest probability of penetration would be likely to occur (*i.e.*, seams and zippers) [38]. One patch was placed on each arm and leg, upper chest, and groin, of each mannequin. One patch was placed in the center of the back opposite the direction of the spray source. A second piece of aluminum foil was placed behind each sample to prevent contact between the sample patch and the Tyvek^®^ suit.

Sample patches were removed from each location with tweezers and placed in 60 mL I-Chem jars with Teflon lids (Thermo Fisher Scientific, Rockwood, TN, USA). Tweezers were rinsed with a 1:1 acetone/toluene solution between each sample to prevent cross contamination. Control samples (two per mannequin) consisted of equivalent sized aluminum squares and were fastened to pieces of cardboard with binder clips at the control site up-wind of the application. Procedures for collecting the control samples followed the same protocol as stated for the bystander mannequins.

Extraction of the tinopal and fluorescein was performed using 15 mL of toluene and deionized water, respectively. Each jar was shaken for 10 s and the liquid was decanted from each jar into a 20 mL analysis vial. Vials were wiped with KimWipes (Kimberly-Clark^®^ Global Sales, LLC, Roswell, GA, USA) to dry the outside of the vials and remove fingerprints before analysis. A GFL-1A fluorometer (Opti-Sciences, Inc., Hudson, NH, USA) was used to detect the amount of light absorbed at a specific wavelength which represented the amount of tracer present in the sample. For the detection of fluorescein the emission filter was 465 nm and the detection filter was 530 nm. For the detection of tinopal OB the emission filter was 370 nm and the detection filter was 430 nm. Absorbance values were recorded for each sample representing deposition of permethrin at each location on the bystander. The detection limit for tinopal and fluorescein is 0.12 and 0.015 ng/cm^2^, respectively. Therefore, based on the amount of insecticide in each formulation the resulting detection limit for permethrin was 0.76 and 0.2 ng/cm^2^, respectively.

Formulations and the order in which the formulations were sprayed were randomly selected each night. A total of 10 applications (replications) of Permanone 30-30 and 10 applications of Aqua-Reslin were performed over the two years. We used R Statistical Package version 2.12.2 (The R Foundation for Statistical Computing, Vienna, Austria) to run analysis of variance (α = 0.05) on log-transformed concentrations to determine differences between location on the body, distances, formulations, and year. Non-detectable concentrations represented less than 10% of the data, so we substituted half of the detection limit for non-detectable concentrations in the data set [39].

## 3. Results and Discussion

**Figure 2.**
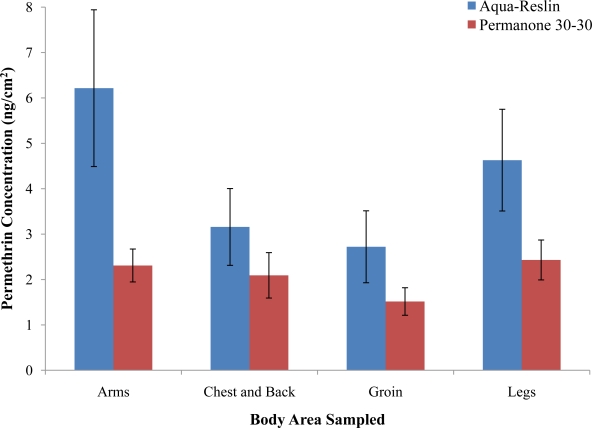
Combined average deposition (±SE) of permethrin for Aqua-Reslin^®^ and Permanone^®^ 30-30 on the arms, chest and back, groin, and legs of bystander mannequins 25 and 50 m from the spray source. No significant differences in dermal deposition of permethrin between the front or back of the mannequins or the placement of the patches on the body. Dermal deposition of permethrin from Permanone 30-30 was significantly less than Aqua-Reslin (*p* = 0.013).

There were no significant differences in dermal deposition of permethrin between the years 2009 and 2010 (*F* = 0.12, *p* = 0.73), distance from the spray source (*F* = 1.64, *p* = 0.21), front or back of the mannequins (*F* = 3.08, *p* = 0.081), or the placement of the patches on the body (*F* = 0.28, *p* = 0.59; Figure 2). However, dermal deposition of permethrin from Permanone 30-30 was significantly less than Aqua-Reslin (*F* = 6.2, *p* = 0.013; Figure 2). Average permethrin concentrations deposited on the body from Aqua-Reslin and Permanone 30-30 were 4.2 and 2.1 ng/cm^2^, respectively.

The greater permethrin deposition of Aqua-Reslin is most likely due to the higher density of the water-based formulation which causes it to settle out faster than the lighter oil-based formulation of Permanone 30-30 [40–42]. Therefore, because of their greater densities, water-based formulations may result in higher exposures to humans than lighter formulations.

Using the assumptions of Schleier III *et al.* [12], the estimated average absorbed dermal exposure to permethrin for an adult male weighing 78.65 kg with head, arms, hands, legs, and feet exposed (surface area = 1.25 m^2^) and a dermal absorption rate of 15% is 0.00005 mg/kg body weight (BW) for Permanone 30-30 and 0.00009 mg/kg BW for Aqua-Reslin (Table 1) [21,36,43]. Schleier III and Peterson [15] measured the average permethrin concentration of 3.3 ng/cm^2^ on deposition pads located on the ground 25 and 50 m from the ground-based ULV applications, which is similar to concentrations measured in the current study. The estimated average absorbed dermal exposure to permethrin estimated by Schleier III [15] was 0.00008 mg/kg BW. Ground-based ULV dermal exposure to permethrin would be 0.0004% of the reference dose, showing that exposures are most likely negligible [21]. The absorption rate of permethrin is based on the technical grade chemical, however the formulation inert ingredients may increase the absorption of permethrin [44].

Our results demonstrate that ground deposition data can be used to estimate potential dermal exposures from ULV applications. However, at distances farther than 50 m deposition concentrations of ground-based ULV applied permethrin have been shown to decrease, which will most likely result in reduced dermal exposure [15]. The absorbed dermal exposures are most likely an overestimation because the U.S. Environmental Protection Agency’s (USEPA) conservative high-end estimate for dermal absorption was used. Recent studies have shown the estimated 24-h dermal absorption rate of permethrin is between 1.2 to 3.3% [21,45,46]. In addition, pyrethroids have a low toxicity to mammals which is attributed to the rapid metabolism in the blood and liver with greater than 90% of pyrethroids being excreted as metabolites in urine within 24 h after exposure [47–50].

Here, we have used passive dosimetry to quantify the dermal exposure of bystanders to ground-based ULV applications. Passive dosimetry has been shown to provide accurate estimates of dermal exposure and to correlate with biomonitoring estimates [51]. The dermal deposition observed in the present study was lower than the concentrations measured by Moore *et al.* [20], which is most likely due to the higher application rate of malathion compared to permethrin. Previous studies of ULV applications have found that 1 to 30% of the insecticide sprayed during application settled onto the ground, with concentrations decreasing substantially over 36 h [15,20,52–55].

Currier *et al.* [56] found no statistical differences in naled, permethrin, and d-phenothrin urinary metabolites in humans from areas that were treated with truck-mounted ULV applications and non-treated areas at application rates of 0.045, 0.002, and 0.004 kg/ha, respectively. Kutz and Strassman [57] and Duprey *et al.* [58] demonstrated that aerial spraying of naled did not result in increased levels of organophosphate urinary metabolites in humans. Other studies have shown that there were no significant increases in asthma related visits to hospitals after ULV applications of pyrethroid insecticides [56–60]. These results, when considered with the risk assessment studies, support that ULV exposures most likely do not result in exposures that exceed a regulatory threshold.

Our results show that dermal exposures to permethrin from ground-based ULV applications are lower than modeled concentrations. In addition, we found that ground deposition of ULV insecticides can be used as a surrogate for estimating dermal exposure. Our results support the findings of previous risk assessments that acute exposures and risks to humans from ULV insecticides are well below regulatory levels of concern.

## Figures and Tables

**Table 1.** Mean permethrin deposition on mannequins in ng/cm^2^ ± standard error, estimated average absorbed dermal exposure mg/kg body weight (BW), and the 95% confidence interval (C.I.) (mg/kg BW) estimated average absorbed dermal exposure for Permanone^®^

**Formulation**	**Concentration (ng/cm^2^)**	**average absorbed dermal exposure (mg/kg BW)**	**95% C.I. average absorbed dermal exposure**
Permanone 30-30	2.1 ± 0.78	0.00005	0.00003–0.00007
Aqua-Reslin	4.2 ± 1.9	0.00009	0.00005–0.00013
